# Meio- and Macrofaunal Communities in Artificial Water-Filled Tree Holes: Effects of Seasonality, Physical and Chemical Parameters, and Availability of Food Resources

**DOI:** 10.1371/journal.pone.0133447

**Published:** 2015-08-18

**Authors:** Christoph Ptatscheck, Walter Traunspurger

**Affiliations:** Animal Ecology, Bielefeld University, Konsequenz 45, 33615, Bielefeld, Germany; Consiglio Nazionale delle Ricerche (CNR), ITALY

## Abstract

**Objectives:**

In this study we investigated the dynamics of meiofaunal and macrofaunal communities in artificial water-filled tree holes. The abundances and, for the first time, biomasses and secondary production rates of these communities were examined. The experimental set-up consisted of 300 brown plastic cups placed in temperate mixed forests and sampled five times over a period of 16 months to determine the impact of (i) seasonal events, (ii) physicochemical parameters, and (iii) food resources on the tree hole metazoans.

**Outcomes:**

Metazoan organisms, especially the meiofauna (rotifers and nematodes) occupied nearly all of the cups (> 99%) throughout the year. Between 55% and 99% of the metazoan community was represented by rotifers (max. 557,000 individuals 100 cm^-2^) and nematodes (max. 58,000 individuals 100 cm^-2^). Diptera taxa, particularly *Dasyhelea* sp. (max. 256 individuals 100 cm^-2^) dominated the macrofaunal community. Macrofauna accounted for the majority of the metazoan biomass, with a mean dry weight of 5,800 μg 100 cm^-2^ and an annual production rate of 20,400 μg C 100 cm^-2^, whereas for meiofauna mean biomass and annual production were 100 μg 100 cm^-2^ and 5,300 μg C 100 cm^-2^, respectively. The macrofaunal taxa tended to show more fluctuating population dynamic while the meiofaunal dynamic was rather low with partly asynchronous development. Seasonality (average temperature and rain intervals) had a significant impact on both meiofauna and macrofauna. Furthermore, bottom-up control (chlorophyll-a and organic carbon), mainly attributable to algae, was a significant factor that shaped the metazoan communities. In contrast, physicochemical water parameters had no evident influence. 23.7% of organism density distribution was explained by redundancy analysis (RDA) indicating a high dynamic and asynchrony of the systems.

## Introduction

Phytotelmata, “plant held waters,” are an inherent part of forest ecosystems, providing small island-like refuges for aquatic and semi-aquatic organisms in an otherwise terrestrial environment. Water-filled tree holes are the globally most common phytotelmata and likely the most frequent type in temperate forests [1,2]. Their high frequency in woodlands, the simple structure of their organismal communities, and their accessibility allow the interactions and processes (e.g., food webs or the influences of specific parameters) of a whole system to be monitored within a relatively small water body, which make phytotelmata highly interesting for ecological research [2,3]. Nevertheless, investigations that include the whole metazoan community, whether within tree holes or other types of phytotelmata, are rare as most studies of these ecosystems have instead focused on the macrofauna (here defined as benthic invertebrates retained on a net with a mesh size of 1,000 μm), including diptera larvae (especially culicids, chironomids, or ceratopogonids) and coleopteran larvae (mostly Scirtidae). All of these organisms are typical inhabitants of water-filled tree holes in temperate forests [2,4,5].

Metazoan communities in tree holes are mainly dominated by rotifers and nematodes, whose dozens of species reach cumulative densities of thousands of individuals cm^−2^ [2,4,6]. However, in aquatic systems the meiofauna (here defined as benthic invertebrates passing a mesh size of 1,000 μm and retained on a net with a mesh size of 40 μm) is an essential link between microbenthos (e.g., bacteria and algae) and macrobenthos and plays an important role in the benthic food web [7–10]. These taxa enter water bodies mainly by wind or rain or via incidental transport by larger animals (e.g., insects) and are able to colonize habitats within a few days [6,11–13]. The ability of nematodes and bdelloidea to undergo anhydrobiosis within a short time enables them to survive environmental extremes (e.g., desiccation or frost) [14–16].

Phytotelmata are highly dynamic systems that are subject to strong seasonal fluctuations [17–19]. Cold seasons but also sustained warm weather may eliminate insect taxa from temporal waters, by frost and desiccation, respectively [17,20,21], or at least delay the development of their larvae [22]. Perturbation by low precipitation reduces macrofaunal diversity, trophic links, and food chain length [23]. Thus, not surprisingly, numerous studies have shown that the water volume in phytotelmata is a crucial physicochemical factor for the survival of insects [20,24,25]. Other physicochemical parameters, such as oxygen content, pH, and conductivity, also shape the macrofaunal composition of these habitats—as evidenced by the preferences of single insect taxa for specific water parameters [5]—and therefore larval development [26]. However, while seasonality and physicochemical parameters are important factors for the macrofauna, they have little if any effect on the meiofauna [4,6].

Due to the lack of macrofaunal top-predators (e.g., odonata larvae or tadpoles) exerting top-down control within Central European phytotelmata, bottom-up effects are particularly important [2,5]. Specifically, the main food source for macrofauna comes from inputs of leaf litter and stem flow, and both are thought to account for the observed bottom-up effects in phytotelmata food webs [1–3]. For example, for insect larvae [23,27] and the meiofauna [6] of Central European phytotelmata, increasing carbon inputs result in an increase in species number, abundance, and trophic links. In tank bromeliads, in addition to detritus, algae may be a relevant resource for the food web [18,28] but its importance as a food source in water-filled tree holes has yet to be investigated.

The importance of local and regional factors has been studied using artificial phytotelmata [6,29–31]. In contrast to their natural analogues, artificial tree holes (cups) provide a standardized experimental setting (e.g., opening diameter and height above the ground), sufficient replicates, and the possibility to exclude specific factors (e.g., leaf litter inputs) [27,32]. In the present study, 300 cups were placed in different temperate mixed-forest sections over a period of 16 months and sampled at five different times.

Our main aims were:

1. to investigate the population dynamics of the whole metazoan community and, for the first time, to examine meiofaunal and macrofaunal abundance, biomass and production within phytotelmata and
2. to define the regulators for the composition of organisms.

In detail, we hypothesized a permanent colonization by meiofaunal taxa showing low seasonal fluctuations due to a continuously random input by passive dispersal their high reproductive rate and the ability to survive adverse natural conditions. In contrast, it can be assumed that macrofaunal organisms whose input is restricted to breeding season might have a fluctuating population dynamic which is significantly affected by seasonal events (e.g., temperature, rainfall) and physicochemical parameters (water volume, oxygen, pH, and conductivity). The food web in phytotelmata is primarily bottom-up controlled. For that reason, we expected that food resources (leaf litter and algae) influence the occurrence and abundance of the community depending on the taxa and therefore shape the metazoan composition in artificial water-filled tree holes.

## Material and Methods

### Experimental sites

**Fig 1:**
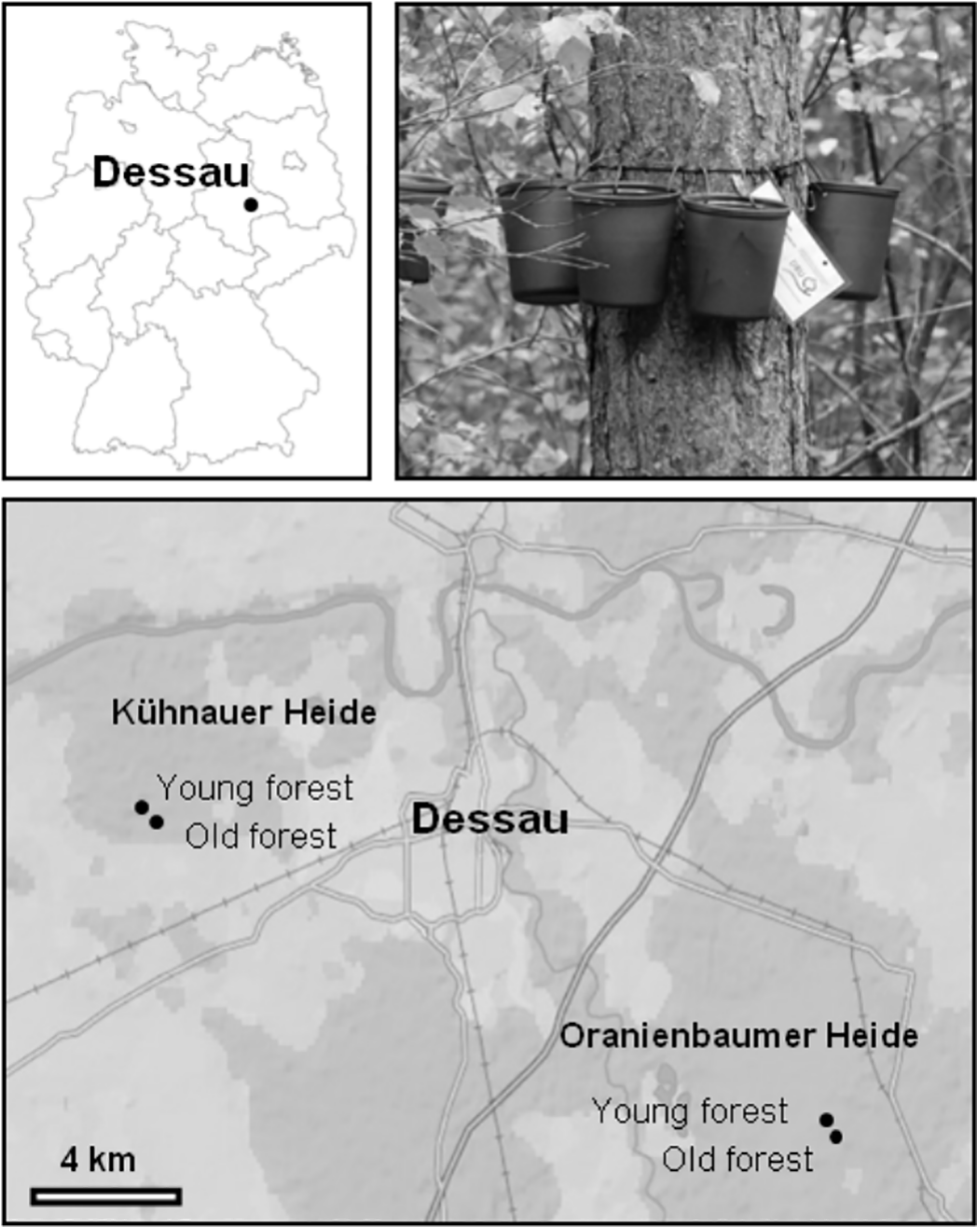
Experimental sites (young and old forest areas) at Kühnauer Heide and Oranienbaumer Heide, located near Dessau (Germany). Artificial tree holes (n = 75), in the form of brown plastic cups, were fixed around the trunks of 15 *P*. *silvestris* (5 cups per tree trunk) at each site.

Our study was conducted between May 2012 and July 2013 at two natural heritage sites, Kühnauer-Heide and Oranienbaumer Heide, near Dessau (Saxony Anhalt, Germany) (Fig 1). Both areas are in possession of the German Federal Environmental Foundation (DBU). Permissions to enter the sites were granted by the public order offices Anhalt-Bitterfeld, Dessau-Rosslau (Kühnauer Heide) and, Wittenberg (Oranienbaumer Heide).

The distance between the two areas is about 20 km. Both sites were army training grounds until 1992. They are characterized by basophile sandy grasslands with patches of heath in their centers, where pioneer woodlands mainly populated by *Betula pendula* Roth and *Pinus sylvestris* L. grow. Along the borders of the two areas are old stocks of *Fagus sylvatica* L., *Quercus robur* L., *Acer campestre* L., and *P*. *sylvestris*. Especially the older tree on both sites contain natural water-filled tree holes. To cover a wide spectrum of forest types, within each heritage site we selected one section (approximately 25 × 25 m) in pioneer woodland (= young forest) adjacent to open land (51°45’59.57’N, 12°22’11.99’E; 51°49’53.84’N, 12°7’4.83’E) and another section, located approximately 600 m away, in an older tree stand (= old forest) (51°45’46.03’N, 12°22’45.23’E; 51°50’2.09’N, 12°7’4.83’E).

### Experimental design

We used brown one-liter polyethylene cups (bottom area 71 cm^2^) as artificial tree holes, as described by Yanoviak and Fincke [31]. At each sampling site, we fixed five cups with polypropylene rope (diameter 4 mm) around the trunk of 15 *P*. *sylvestris* individuals at 2 m above the ground (= 300 cups, 15 trees × 5 cups × 2 sites × 2 forest types) (Fig 1). Additionally, we filled the cups with 500 ml of distilled water. The cups had direct contact with the tree bark, which allowed stem-flow inputs. Our experiment was started in March 2012. We obtained the meteorological data (rainfall and temperature) for each day of the experiment from a weather station in Dessau (51°47‘55‘N, 12°14‘46‘E; about 10 km away from the sampling sites).

### Sampling

We sampled the cups in May, August, and November of 2012 and in March and July of 2013. For this purpose we removed one cup from each tree (number of replicates = 15) per sampling time. We measured the volume, pH, O_2_ content, and conductivity of the water in each cup in the field with probes (Hanna HI 9828). When the water was frozen (March 2013), we performed the measurements (water volume, pH, and conductivity) in the laboratory after the water had thawed at room temperature. We rinsed each cup using a washing bottle to collect all organisms and algae from the inner walls. We carried out the whole sampling process of a sampling date for all sites within one day.

In the laboratory, we brought the volume of each sample to one liter with water and abstracted 30 ml each (containing organisms, detritus, leaf litter and other organic material) with a sample divider for ash-free dry mass (AFDM) and chlorophyll-a (Chl-a) measurements. We chopped the particles within the sampling divider to enable a homogeneous separation. Finally, we sieved the remaining volume (1-cm mesh size) to remove coarse particles, stained the samples with Rose Bengal, and preserved them in 37% formaldehyde (final concentration 4%).

### Chl-a content and AFDM

We measured the Chl-a content (μg cm^-2^), as a proxy of algal biomass. For this purpose we filtered the 30-ml subsamples onto glass-fiber-filters (Whatman; 25 mm diameter), which were stored at -18°C until the analysis. We extracted the Chl-a with ethanol (90%) at 4°C in the dark for 24 h. For the measuring and determining of Chl-a concentrations, we used spectrophotometry and the pheophytin-uncorrected values [33].

To obtain the AFDM as indicator of organic material, we filtered 15 ml of each subsample onto pre-combusted (550°C, 7 h) glass-fiber-filters (Whatman; 25 mm diameter). We dried the filters for 24 h at 105°C and then combusted them for 7 h at 550°C. Subsequently, we determined the AFDM (mg cm^−2^) based on the differences in weight.

### Meiofaunal and macrofaunal abundances, classification, biomass and secondary production rates

For determination of the meiofaunal (nematodes, rotifers, and tardigrades) and macrofaunal (different dipteran larvae and coleopteran larvae) abundances in subsamples, we used a LEICA L2 stereo-microscope (40× magnification). We identified the insect larvae at least to the family level and considered the organisms contained in the extracted volumes for AFDM and Chl-a measurements.

For biomass (dry weight) calculations, we measured the lengths, heights, and widths of 50 nematodes, rotifers, and tardigrades and the lengths of all insect larvae in each sample. We grouped all taxa into specific size classes (Table 1). In addition, we dried 40 measured *Myathropa* sp. larvae for 24 h at 80°C. We determined their dry weights by fitting the power function described by Benke et al. [34] to obtain the values of a and b. The specific methods used in the biomass calculations of the different taxa are listed in Table 1.

**Table 1:** Size classes and methods used for calculating the biomass as dry weight (DW).

Taxon	Size classes (mm)	Method	×Reference
Nematodes	<0.25; 0.25-<0.5;…3.75-<4	DW^a^	[36,37]
Bdelloidea	< 0125; 0.125-<0.25; 0.25-<0.5	DW^b^	[37,38]
Tardigrades	0.125-<0.25; 0.25-<0.5	DW^b^	[37,38]
*Dasyhelea* sp.	<0.5; 0.5-<1;…6.5-<7	DW^c^	[34]
*Metriocnemus* sp.	<0.5; 0.5-<1;…14.5-<15	DW^c^	[34]
Muscidae	0.25-<0.5;…6-<6.5	DW^c^	[34]
*Psychodidae* sp.	4-<4.5; 4.5-<5; 5-<5.5	DW^c^	[34]
*Cheilosia* sp.	0.25-<0.5;…6-<6.5	DW^c^	[34]
Tabanidae	1-<1.5;…6-<6.5	DW^c^	[34]
Scirtidae	1-<1.5;…22.5-<23	DW^c^	[34]
Culex sp.	1-<1.5;…10-<10.5	DW^d^	[39]
Myathropa sp.	1.5-<2;…21.5-<22	DW^e^	[34]

^a^ Width^2^ × length/1600000, using the specific gravity (1.13 g cm^−3^) and a dry/wet weight ratio of 0.25

^b^ 0.8×length×width*height, assuming a dry/wet weight ratio of 0.25

^c^ a × length^b^, where a and b are specific for the respective taxon

^d^ 4.4×10^3^*e^0.8*length^

^e^ a × length^b^, where a = 0.0024 and b = 3.0651

To obtain a comprehensive description of metazoan community, we calculated the abundances and biomasses as percentages and ratios (meifauna:macrofauna). The resulting descriptions were not necessarily congruent because of the large variances in abundances and the fact that some of the cups were settled only by meiofauna or macrofauna.

We estimated the mean annual secondary production (g m^−2^) based on the mean biomass (B, g m^−2^) of the single taxa, the maximal individual biomass per taxon (M_max_, mg individual^−1^) and the mean annual daily temperature (T, 10.1°C), according to Plante and Downing [35]:

$$\text{Log}\;\left(\text{production}\right)=0.06+0.79^{\;*}\;\text{Log}\left(\text{B}\right)-0.16^{\;*}\;\text{Log}\left({\text{M}}_{\text{max}}\right)+0.5^{\;*}\;\text{T}$$ (1)

### Statistical analysis

We applied a canonical ordination analysis (CANOCO, version 4.5) to log_(x+1)_-transformed data to assess the influence of environmental factors on the density distribution of meiofaunal and macrofaunal organisms in the artificial tree holes over the five sampling times. We regarded Chl-a content, AFDM, O2 content, conductivity, pH, water volume, days after last rain, average daily rain, days after last frost and average daily temperature in the analysis.

Because the total inertia (1.47) measured by a detrended correspondence analysis (DCA) was < 2.6, we expected a predominance of linear group response curves [40]. Therefore, we used a redundancy analysis (RDA), in which the ordination axes were constrained to be linear combinations of abiotic and biotic factors, to investigate the relationships between these factors and the distribution of taxa. We listed the factors (conditional effects) according to the variances they explained individually (i.e., without eventual co-variability with other factors), as given by their eigenvalues (λ). To test the statistical significance of the factors we used Monte Carlo permutations (999 unrestricted permutations, α = 0.05).

Additionally, we performed Kruskal-Wallis tests to analyse the impact of the sampling date on the abundances of the most common metazoan taxa.

## Results

### Metazoan population dynamics

From the initial 300 cups, 264 were examined; the remainders were lost during the field trial due to frost, desiccation, and thunderstorms. The number of replicates per sampling site that were finally investigated and the respective sampling times are listed in S1 Table. In March the water was frozen whereas in May the contents of the cups were dried out to near desiccation. Nonetheless, all of the artificial tree holes were settled by meio- and macrofaunal organisms throughout the year, with the exception of two cups from the March 2013 sampling, in which no metazoans were found.

Twelve different taxa colonized the artificial tree holes (S1 Table). The meiofauna (rotifers, nematodes, and tardigrades) consisted of passively dispersed organisms. The macrofauna was represented by one coleoptera taxon (Scirtidae) and eight diptera taxa (*Dasyhelea* sp., *Metriocnemus* sp., *Culex* sp., *Myathropa* sp., *Cheilosia* sp., Muscidae, Tabanidae, and *Psychoda* sp.).

**Fig 2:**
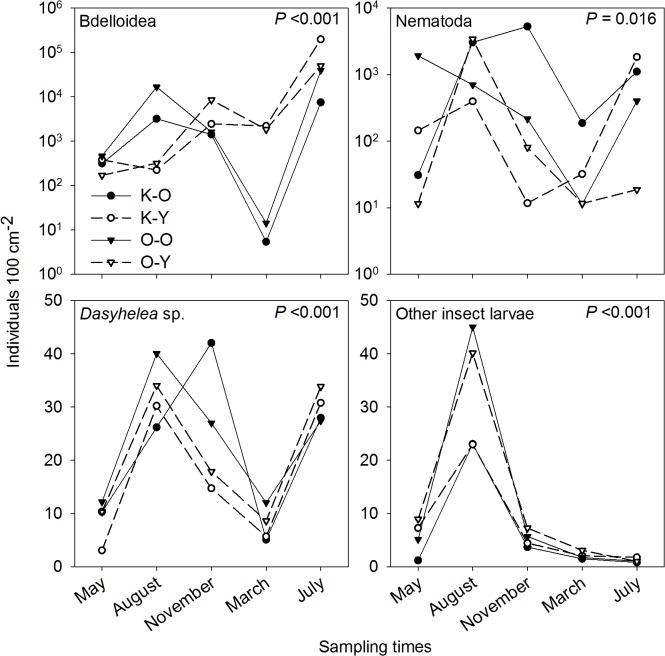
Mean abundances of Bdelloidea, Nematodes, and *Dasyhelea* sp. and other insect larvae per 100 cm^2^. The SD’s are listed in S1 Table. The data are given for the four sampling sites, K-O (Kühnauerheide, old forest), K-Y (Kühnauerheide, young forest), O-O (Oranienbaumer Heide, old forest), and O-Y (Oranienbaumer Heide, young forest), at the five sampling times. Note the differences in the y-axes. The p-values (Kruskal-Wallis test) indicate the overall impact of the sampling date on the taxon.

The most abundant organisms in the cups were bdelloid rotifers, which had a mean density of 190,000 ind 100 cm^2^ (maximum density: 557,000 ind 100 cm^2^) in the young forest of Kühnauer Heide in July (Fig 2). Rotifer abundances varied greatly between the cups of a single sampling time and among the four different sites. At least 82% of the artificial tree holes were occupied by bdelloid rotifers during March, August, and November 2012 and July 2013. In March 2013, fewer cups were settled (55–77%) and the mean number of bdelloid rotifers in the cups of the old forests declined to less than 15 individuals per 100 cm^2^.

Nematodes colonized 91% of the cup-contained water bodies. The mean abundance widely varied, between 11 ind 100 cm^−2^ and 5,280 ind 100 cm^−2^, with higher densities during summer (August 2012 and July 2013, Fig 2). In single replicates, nematode densities were as high as 58,000 individuals per 100 cm^2^. Among the macrofaunal taxa, *Dasyhelea* sp. (Ceratopogonidae) was the most common and abundant, as it settled in a mean 67% (± 23.6% SD) of the surveyed cups and its mean density was in the range of 3–42 ind 100 cm^−2^, reaching a maximum abundance of 256 individuals 100 cm^−2^. In May 2012 and March 2013, *Dasyhelea* sp. densities were lowest (Fig 2). All other insect taxa in the artificial tree holes were represented by only a few (< 5) individuals per cup (Fig 2), with the exception of Muscidae larvae, which in August 2012 showed mean abundances of 16–39 individuals 100 cm^−2^.

**Fig 3:**
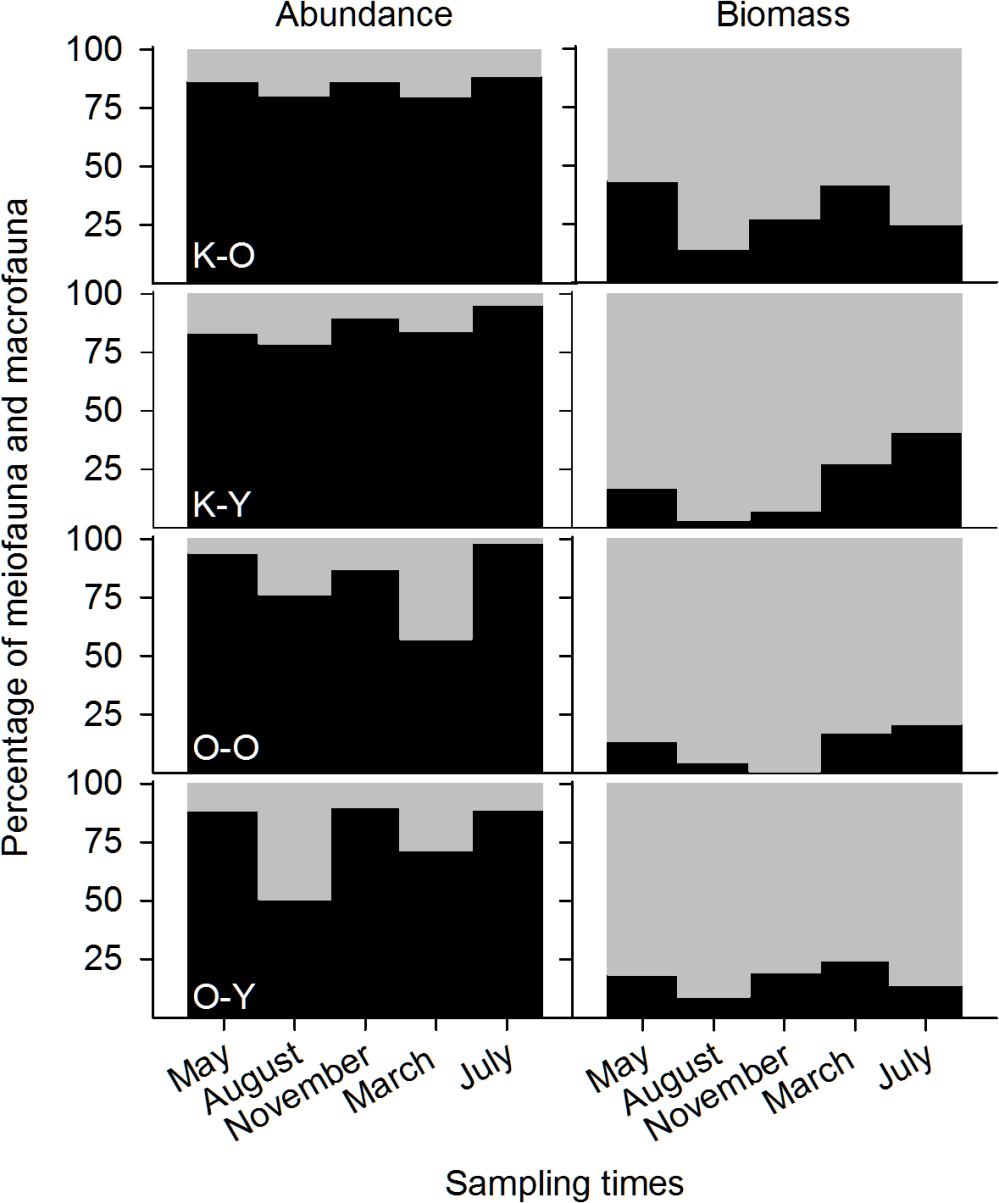
Mean percentages of meiofaunal (black areas) and macrofaunal (gray areas) abundances (left column) and biomasses (right column). Data are given for the four sampling sites, K-O (Kühnauerheide, old forest), K-Y (Kühnauerheide, young forest), O-O (Oranienbaumer Heide, old forest), and O-Y (Oranienbaumer Heide, young forest), at the five sampling times.

The metazoan community was strongly dominated by meiofauna (83.4% ± 26.4%, mean ±SD) (Fig 3), with mean densities of 15,000 ind 100 cm^−2^ (± 60,000 ind 100 cm^−2^ SD) compared to 31 ind 100 cm^−2^ (± 46 ind 100 cm^−2^ SD) for the macrofauna. In August 2012 and March 2013, meiofaunal percentages declined at all sampling sites to 51%. By contrast, during these months macrofauna represented the major part of metazoan biomass (81% ± 33.9%, mean ± SD) (Fig 3), with mean dry weights of 5,800 μg 100 cm^−2^ (± 14,600 μg 100 cm^-2^ SD) compared to 100 μg 100 cm^−2^ (± 400 μg 100 cm^−2^ SD) for meiofauna (S2 Table). Annual secondary production was 20,400 μg C 100 cm^−2^ and 5,300 μg C 100 cm^−2^ for macrofauna and meiofauna, respectively. The taxa with the greatest contribution to production were *Myathropa* sp. (42.7%), nematodes (16.1%), and *Dasyhelea* sp. (13.7%). The most abundant group, rotifers, contributed 4.5%.

### Factors impacting the organism density distribution

A RDA analysis was performed for the 264 artificial tree holes from the four forest sites to investigate the influence of seasonal effects (rain, drought, temperature), physiochemical factors (O_2_, water volume, conductivity, pH) and food resources (Chl-a and AFDM) (S3 Table) on community composition and taxonal abundances. Chl-a (λ 0.15), the number of days after the last rain event (λ 0.04), AFDM (λ 0.02), and the average daily temperature (λ 0.02) had the most significant impacts (Monte Carlo permutation test, p < 0.05) on organismal density distribution (Table 2).

**Table 2:** Results of a redundancy analysis testing the effects of environmental factors on the density distribution of invertebrate taxa in 264 artificial tree holes.

Marginal effects		Conditional effects		
Variables	λ	Variables	λ	P
Chl-a	0.15	**Chl-a**	**0.15**	**0.001**
Avg. daily rain	0.09	**Last rain**	**0.04**	**0.001**
AFDM	0.07	**Avg. daily temperature**	**0.02**	**0.005**
Last rain	0.06	**AFDM**	**0.02**	**0.017**
Avg. daily temperature	0.06	O_2_ content	0.01	0.158
Last frost	0.05	Avg. daily rain	0	0.145
O_2_ content	0.03	Last frost	0	0.274
Water volume	0.01	Conductivity	0	0.768
Conductivity	0			
pH	0			
		**Sum of all λ**	**0.24**	

The factors are listed by their eigenvalues (λ). For the calculation of p-values we used the Monte Carlo permutation test (999 iterations).

Axis 1 of the RDA explained 20.8% of the organism density distribution (species environmental correlation = 0.59), based on the positive correlation with Chl-a and AFDM (Table 3). Axis 2 explained 2.1% of the organismal density distribution, reflecting a negative correlation with the number of days after the last rain and positive correlations with the averaged daily rain and the number of days after the last freezing event. Overall, 23.7% of the organismal density distribution was explained by the RDA. None of the other physicochemical factors (O_2_ content, pH, conductivity) had a significant impact on the metazoans, and nor did water volume.

**Table 3:** Statistical summary of the redundancy analysis of 264 artificial tree holes and seven environmental factors.

Axes	1	2	3	4	Total variance
Eigenvalues	0.208	0.021	0.004	0.002	1.0
Species-environment correlations	0.588	0.385	0.164	0.167	
Cumulative percentage variance of					
Species data	20.8	23	23.4	23.6	
Species-environment relation	88.6	97	99.9	99.7	
Sum of all eigenvalues					1.0
Sum of all canonical eigenvalues					0.24

**Fig 4:**
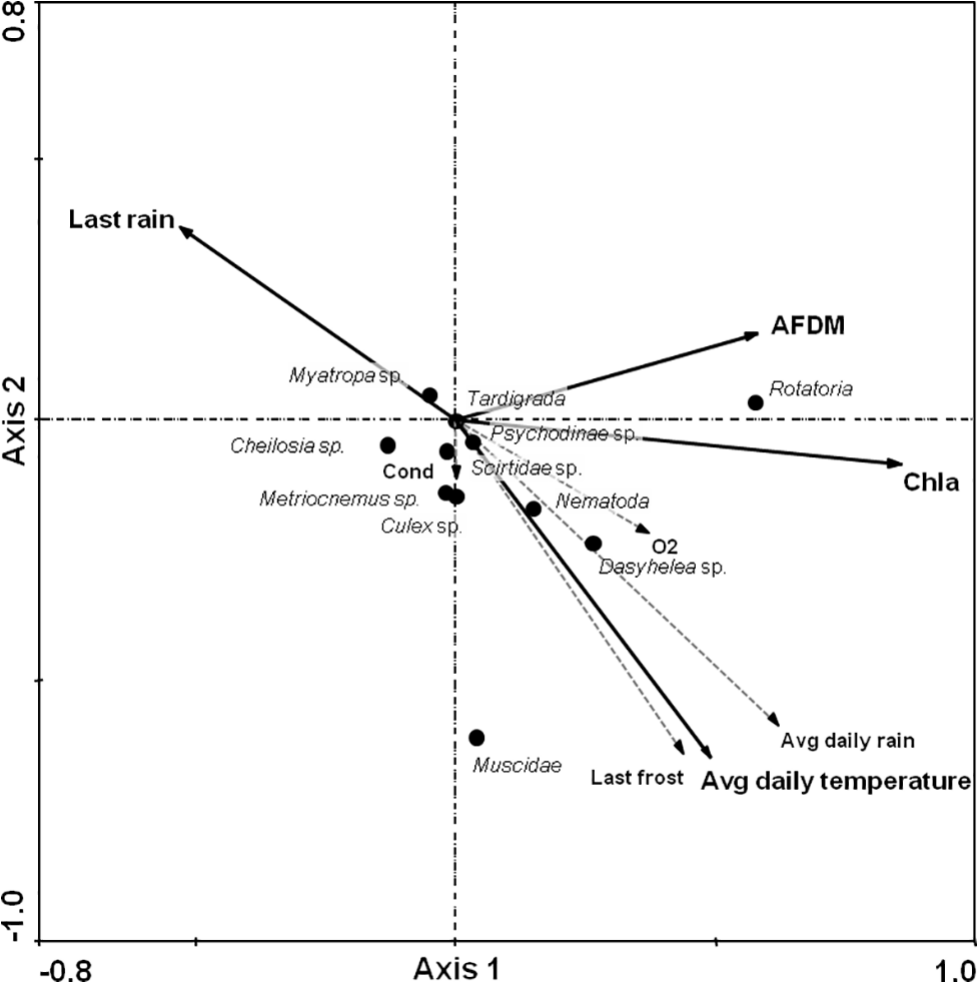
Redundancy analysis biplot of the 12 identified taxa and eight relevant environmental factors. Significant predictors of the organism density distribution are shown in bold (Monte Carlo permutation test, p < 0.05). Abbreviations: Last rain = number of days after the last rain; Last frost = number of days after the last frost; Chla = chlorophyll a; Cond = conductivity. Sum of all canonical eigenvalues: 0.237 (axis 1: 0.208; axis 2: 0.021).

Thus, in the interpretation of the RDA biplot shown in Fig 4, for the organisms on the right side the density distributions were positively influenced by bottom up effects (algae and AFDM) and, especially in the lower right corner, the higher temperature and rainy conditions.

Rotifers, which represented the major part of the metazoan community in the tree holes, clustered together with Chl-a and AFDM. Nematodes and *Dasyhelea* sp. were also located on the right side of the plot but with a wider spread, and more closely linked to higher temperature and more frequent precipitation. Muscidae scored in the lower right area, near the zero-point of axis 1, indicating no affinity for larger amounts of resources. Unlike the other frequent taxa, both Syrphidae *Cheilosia* sp. and *Myatropa* sp. tended to prefer lower temperatures and dryer environments, respectively, and were negatively impacted by greater resource availability.

**Fig 5:**
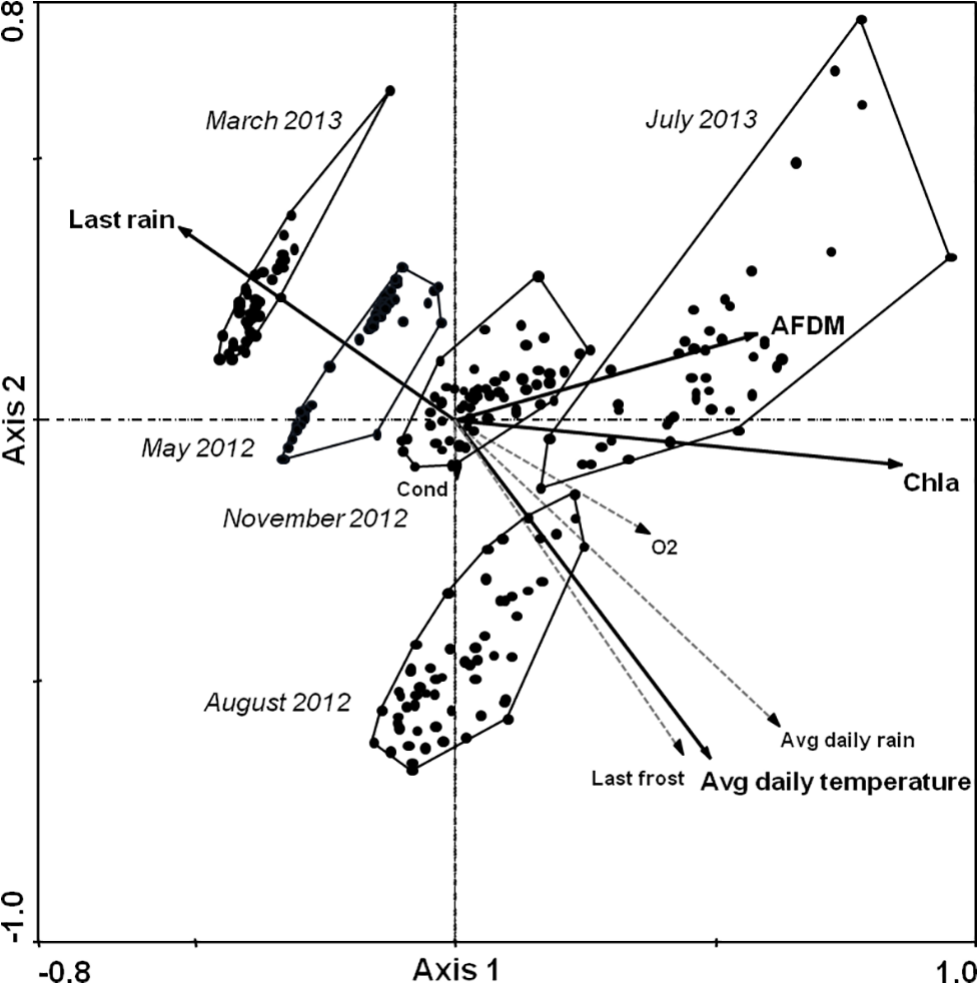
Redundancy analysis biplot of the 264 artificial tree hole communities and eight environmental factors. Significant predictors of the organismal density distribution are shown in bold. Samplings made on the same date are framed. For an explanation of the environmental factor abbreviations, see Fig 2. Sum of all canonical eigenvalues: 0.237 (axis 1: 0.208; axis 2: 0.021).

During the study period, there were no verifiably differences between the four sampling sites with respect to organismal density distributions, whereas clear seasonal influences were observed. The Kruskal-Wallis tests indicated significant impact (*P* < 0.016) of the sampling date on all common taxa (bdelloidea, nematoda, *Dasyhelea* sp. and the other insect larvae) (Fig 2). In the RDA biplot the 264 samples clustered within the five sampling times and were well separated (Fig 5). Samples from May 2012 and March 2013, when temperature and rainfall were lowest, placed in the upper left of the RDA biplot whereas samples from August 2012 and July 2013 plotted roughly ahead of resource availability and warmer conditions. The November 2012 samples scattered around the zero point and along with AFDM.

## Discussion

We documented the development of the metazoan community in artificial water-filled tree holes within a temperate forest ecosystem over a period of 16 months. All of the taxa detected during the field trial and the values of the physicochemical parameters corresponded to those reported in studies of natural water-filled tree holes from central Europe [2,4,5].

There have been only a few comprehensive studies of freshwater ecosystems in which both meiofaunal and macrofaunal abundance, biomass, and secondary production were documented. The metazoan abundances in the artificial tree holes were certainly within the scope of similar studies on lentic [41–43] and lotic [44] waters. The clear predominance of meiofauna resulted in a meiofauna:macrofauna ratio of 491:1. While Anderson and De Henau [41] and Nalepa and Quigley [42] determined ratios typically between approximately 10:1 and 80:1 for different lakes, single deviations up to 812:1 were recorded. In our study, the high densities of rotifers, up to 56 million individuals m^−2^ (mean 20 millions m^−2^), shows that the artificial tree holes can be quickly colonized by rotifers, but based on densities up to 5.8 million m^−2^, nematodes can also be considered as good colonizers. In fact, only in the periphyton of three Swedish lakes, in the sediment of the volcanic lake in Galapagos, and in the sediment of Lake Constance were a greater number of nematodes recorded [10,45,46].

By contrast, in the artificial tree holes, metazoan biomass, and especially meiofaunal biomass (accounting in some samples for < 10%), was relatively low. This amount is consistent with observations in larger water bodies [9,41,42]. The calculated dry weight biomass of the meiofauna and macrofauna in the artificial tree holes was 10 mg m^−2^ and 584 mg m^−2^, respectively. The ratio of macro- to meiofauna was 1:55. In Lake Brunnsee, meiofaunal and macrofaunal biomass was 110 mg m^−2^ and 1180 mg m^−2^, respectively, resulting in a ratio of 1:11. This study as well as those of Ptatscheck and Traunspurger [6] and Devetter [4] demonstrated that the meiofauna in water-filled tree holes in Middle Europe mostly consists of rotifers and nematodes (> 90%). However, the contribution of rotifers to biomass is much smaller than that of organisms such as oligochaetes, copepods, and ostracodes [43,47]. These taxa are known to colonize tree holes in Europe but their main distribution ranges are the tropics (reviewed by Kitching, [2]), where meiofaunal biomasses are likely to be accordingly higher.

Bergtold and Traunspurger [9] calculated annual meiofaunal and macrofaunal production rates of 1.6–1.7 g C m^−2^ and 3.1–6.7 g C m^−2^. These values correspond to a ratio of macro- to meiofauna between 1:2 and 1:4. In this study, annual secondary production in water-filled tree holes was 0.58 g C m^−2^ for meiofauna and 2.04 g C m^−2^ for macrofauna. Both values are roughly one-third of the production in Lake Brunnsee [9], but the ratio (1:3.5) is very similar.

Interestingly, the contribution of nematodes to total annual metazoan secondary production (16.1%) was quite high and in contrast to the < 3.5% reported for nematodes in larger aquatic systems [44,48]. Thus, the importance of meiofauna to the metazoan community in phytotelmata has clearly been underestimated.

Secondary production rates will differ depending on the method used. The numerous approaches can generate misleading results (reviewed by [49,50]). It is also possible that in other types of phytotelmata (e.g., tank bromeliads or pitcher plants) meiofaunal and macrofaunal composition, and therefore secondary production, will differ. Nonetheless, this study is the first to provide basic information on the metazoan community in water-filled tree holes and comparisons with other aquatic systems.

In accordance with our hypothesis the results clearly show that seasonality effects (related to temperature and precipitation), on the one hand, and bottom-up effects, on the other, were the fundamental factors that shaped the metazoan communities within the artificial tree holes. In contrast to our expectation, not only the macrofauna but also the meiofauna were significantly influenced by season.

Additionally, both the metazoan density distribution and the percentage of meiofauna and macrofauna indicated synchronous, seasonal shifts in both forest areas. This was particularly the case for insects. Temperature-induced extremes such as almost total desiccation or frost reduced both the number of cups settled by single insect taxa (up to their elimination) and insect abundances, as reported previously [4,21,22]. The annual population dynamics of nematodes and rotifers tended to be low and in some cases were even asynchronous, consistent with the observations of Devetter [4] and confirming our hypothesis. Although some of the most common taxa temporarily disappeared over the course of the study period, the cups were almost continuously colonized by metazoans especially by meiofaunal taxa as we had expected. This observation has important implications for small bodies of water in terms of their subsequent recolonization [21].

Physicochemical parameters (water volume, oxygen, pH, and conductivity), however, had no important influence on these metazoans, not even on the macrofauna as initially assumed.

It should be noted that the standardized experimental design using artificial tree holes might led to a low variance of the physicochemical parameters in comparison to natural analogues. For that reason the observed values were possibly too narrow to capture conditions that might impact metazoan.

In our study, it was not the water volume, as often shown [20,24,25], but rather the interval between rain events that was decisive in determining the organism density distribution. Stem-flow, defined as rain water running down the tree surface, contains small metazoans, bacteria, algae, and nutrients that are washed into tree holes, thereby enriching their communities or serving as resources [11,26,51]. In addition, the flushing effects of stem-flow affect the milieu already established within the phytotelmata [52]. Indeed, our RDA analysis showed that Chl-a content was higher with shorter rain intervals, suggesting an input by precipitation. Furthermore, the Chl-a content changed during the 16 months of the study, with declines caused by freezing and drought. Both Chl-a content and AFDM were highest in July 2013, coinciding with the densities of Bdelloidea and *Dasyhelea* sp.

This is the first study to show that, along with the amount of leaf litter, algae are an important food resource in the water-filled tree holes of temperate forests, as previously reported for tank bromeliads [18,28,53]. As a potential algal feeder, bdelloidea correlated well with Chl-a content [54,55] (Fig 3).

Among the insect fauna found in middle European tree holes, saprophagous species, feeding on leaf litter, are the most common [2,5]. Both the leaf litter itself and its breakdown products promote the growth of bacteria and fungi which are consumed by the insect larvae and bacteriovorus nematodes that predominate in water-filled tree holes [6,18,28,52]. Thus, the abundance, diversity, and number of trophic levels of macrofauna and meiofauna increased with higher amounts of leaf litter [6,23]. This is not the case for the syrphid larvae of *Myathropa* sp. and *Cheilosia* sp., which, as previously noted by Schmidl et al. [5], seem to prefer less productive containers.

The variances in organismal abundances and in local parameters were unexpectedly high given that the artificial tree holes were initially identical. Indeed, 23.7% of the organismal density distribution could be explained by a multivariate statistic. The remaining, unexplained variance is indicative of the wide-ranging dynamics of these small aquatic systems and the asynchronous development of phytotelmata communities [17–19].

Other important factors shaping the composition of phytotelmata communities are colonization events [17]. Thus, while adult insects mainly respond to the breeding season in the placement of their eggs, nematodes and rotifers enter these small refuges continuously, deposited by wind and rain [11–13]. Small organisms (e.g., rotifers) are fast and ubiquitous dispersers without spatial limitations [56,57] while the asynchronous changes in nematode abundances (Fig 2) suggest random colonization

Although our study did not include an analysis of predator-prey interactions between meiofauna and macrofauna, that both rotifers and nematodes serve as prey for insect larvae has been reported in several studies [7,28,58,59]. While an examination of meiofaunal population dynamics determined on a species level can reveal the impact of colonization, as achieved in this study, the top-down influence of the macrofauna on the meiofauna remains to be investigated.

Nonetheless, our study provides a comprehensive documentation of the meiofauna and macrofauna that inhabit artificial water-filled tree holes over the course of the year and a preliminary comparison of metazoan biomass and secondary production between this and other aquatic systems. We demonstrated a synchronous development for the macrofaunal organisms while the meiofauna that colonized nearly all cups showed rather asynchronous and less fluctuating population dynamic. However, we also identified seasonality (daily temperature and rain intervals) and bottom-up effects as fundamental factors that determine organismal density distribution of the whole meatazoans. While leaf litter is well known to be the main food source in phytotelmata, we identified algae as a second food-web pathway. The measured physicochemical parameters had no significant impact on the meiofauna and surprisingly not even on the macrofauna. Our investigation contributes to a better understanding of metazoan communities in the phytotelmata within temperate forests.

## Supporting Information

S1 Table Average organism abundances (±SD) per 100 cm^2^. K-O (Kühnauerheide, old forest), K-Y (Kühnauerheide, young forest), O-O (Oranienbaumer Heide, old forest), O-Y (Oranienbaumer Heide, young forest).
(DOC)

S2 Table Average organism biomass (μg 100 cm^-2^) (±SD). K-O (Kühnauerheide, old forest), K-Y (Kühnauerheide, young forest), O-O (Oranienbaumer Heide, old forest), O-Y (Oranienbaumer Heide, young forest).
(DOC)

S3 Table Average (±SD) physicochemical parameters and meteorological data. K-O (Kühnauerheide, old forest), K-Y (Kühnauerheide, young forest), O-O (Oranienbaumer Heide, old forest), O-Y (Oranienbaumer Heide, young forest); * Because of the low water level the probes could only be used in single cups such that a mean could not be determined. Chl-a, chlorophyll-a; AFDM, ash-free dry mass.
(DOC)
