# Supplementary material for: Meio- and Macrofaunal Communities in Artificial Water-Filled Tree Holes: Effects of Seasonality, Physical and Chemical Parameters, and Availability of Food Resources
Source: PLoS One. 2015 Aug 18;10(8):e0133447. doi: 10.1371/journal.pone.0133447 (PMC4540321; doi:10.1371/journal.pone.0133447)
Supplement: S1 Table — K-O (Kühnauerheide, old forest), K-Y (Kühnauerheide, young forest), O-O (Oranienbaumer Heide, old forest), O-Y (Oranienbaumer Heide, young forest). (DOC) [file pone.0133447.s001.doc]

| Sample | |  | Average abundance per 100cm² | | | | | | | | | | | |
| --- | --- | --- | --- | --- | --- | --- | --- | --- | --- | --- | --- | --- | --- | --- |
|  |  |  | Nematoda | | Bdeloidea | | Tardigrada | | *Dasyhelea sp.* | | *Metriocnemus sp.* | | *Culex sp.* | |
| **May 2012** | **K-O** | n=15 | 30.8 | (±68.6) | 313.9 | (±538.0) | 0.1 | (±0.4) | 10.3 | (±15.5) |  |  |  |  |
|  | **K-Y** | n=15 | 144.5 | (±347.1) | 377.4 | (±699.7) | 0.3 | (±0.8) | 3.0 | (±7.8) |  |  |  |  |
|  | **O-O** | n=15 | 1,924.0 | (±6,779.7) | 460.0 | (±425.5) |  |  | 12.2 | (±19.7) |  |  |  |  |
|  | **O-Y** | n=15 | 11.5 | (±15.8) | 169.2 | (±228.1) |  |  | 10.3 | (±39.9) |  |  |  |  |
| **August 2012** | **K-O** | n=15 | 3,064.8 | (±4,861.8) | 3,192.1 | (±6,188.7) |  |  | 26.2 | (±45.8) |  |  |  |  |
|  | **K-Y** | n=15 | 396.0 | (±715.3) | 222.8 | (±183.8) |  |  | 30.2 | (±31.9) | 3.7 | (±12.9) |  |  |
|  | **O-O** | n=15 | 700.9 | (±2,680.0) | 16,561.2 | (±33,862.2) | |  | 40.0 | (±54.4) |  |  | 0.1 | (±0.4) |
|  | **O-Y** | n=14 | 3,401.0 | (±11,337.3) | 316.3 | (±568.3) |  |  | 34.1 | (±31.0) | 0.4 | (±1.2) | 0.8 | (±2.1) |
| **November 2012** | **K-O** | n=14 | 5,280.4 | (±15,446.3) | 1,413.5 | (±4,101.4) |  |  | 42.0 | (±77.1) |  |  |  |  |
|  | **K-Y** | n=15 | 11.7 | (±11.3) | 2,445.2 | (±5,748.6) |  |  | 14.7 | (±33.2) |  |  |  |  |
|  | **O-O** | n=15 | 214.6 | (±702.5) | 1,623.2 | (±2,969.6) |  |  | 27.0 | (±29.3) |  |  |  |  |
|  | **O-Y** | n=14 | 80.3 | (±230.8) | 8,493.0 | (±13,108.7) | |  | 17.9 | (±24.7) |  |  |  |  |
| **March 2013** | **K-O** | n=9 | 186.5 | (±520.2) | 5.4 | (±6.3) |  |  | 5.1 | (±6.6) |  |  |  |  |
|  | **K-Y** | n=13 | 32.0 | (±91.9) | 2,201.0 | (±6612.3) |  |  | 5.7 | (±7.7) |  |  |  |  |
|  | **O-O** | n=6 | 11.3 | (±21.9) | 14.2 | (±26.5) |  |  | 12.0 | (±18.7) |  |  | 0.2 | (±0.6) |
|  | **O-Y** | n=13 | 11.5 | (±19.6) | 1,823.8 | (±5,860.9) |  |  | 8.6 | (±18.3) |  |  |  |  |
| **July 2013** | **K-O** | n=13 | 1,108.1 | (±2,343.7) | 7,498.1 | (±20,050.0) | 0.3 | (±0.9) | 28.0 | (±40.6) | 0.2 | (±0.8) |  |  |
|  | **K-Y** | n=11 | 1,834.7 | (±5,956.0) | 19,7592.1 | (±18,6913.1) | |  | 30.8 | (±41.9) |  |  |  |  |
|  | **O-O** | n=13 | 401.4 | (±1,197.0) | 39,431.6 | (±85,854.5) | |  | 27.4 | (±29.4) |  |  |  |  |
|  | **O-Y** | n=9 | 18.6 | (±32.7) | 50,040.8 | (±73,042.5) | |  | 33.8 | (±25.2) | 0.3 | (±0.6) |  |  |
|  |  |  |  |  |  |  |  |  |  |  |  |  |  |  |

| Sample | |  | Average abundance per 100cm² | | | | | | | | | | | |
| --- | --- | --- | --- | --- | --- | --- | --- | --- | --- | --- | --- | --- | --- | --- |
|  |  |  | Muscidae | | *Psychodidae sp.* | | *Myathropa sp.* | | *Cheilosia sp.* | | Tabaindae | | Scirtidae | |
| **May 2012** | **K-O** | n=15 | |  |  |  |  |  | 1.1 | (±1.3) |  |  | 0.1 | (±0.4) |
|  | **K-Y** | n=15 | |  |  |  |  |  | 6.9 | (±7.5) |  |  | 0.4 | (±1.2) |
|  | **O-O** | n=15 | |  |  |  |  |  | 2.9 | (±2.6) |  |  | 2.2 | (±5.2) |
|  | **O-Y** | n=15 | |  |  |  |  |  | 7.2 | (±6.1) |  |  | 1.8 | (±3.1) |
| **August 2012** | **K-O** | n=15 | 20.4 | (±42.1) |  |  |  |  | 2.2 | (±2.4) | 0.1 | (±0.4) | 0.4 | (±0.9) |
|  | **K-Y** | n=15 | 16.1 | (±17.6) |  |  |  |  | 2.9 | (±5.8) | 0.1 | (±0.4) | 0.1 | (±0.4) |
|  | **O-O** | n=15 | 38.9 | (±79.8) | 1.4 | (±5.3) | 0.6 | (±1.2) | 2.9 | (±2.7) | 0.3 | (±0.6) | 0.8 | (±1.2) |
|  | **O-Y** | n=14 | 31.4 | (±29.9) |  |  | 0.6 | (±1.0) | 4.6 | (±9.5) | 0.5 | (±1.4) | 1.7 | (±2.3) |
| **November 2012** | **K-O** | n=14 | 1.79 | (±1.7) |  |  | 0.8 | (±2.0) | 0.8 | (±1.1) | 0.2 | (±0.5) |  |  |
|  | **K-Y** | n=15 | 2.35 | (±1.7) |  |  | 0.4 | (±0.7) | 1.5 | (±2.6) | 0.1 | (±0.4) | 0.1 | (±0.4) |
|  | **O-O** | n=15 | 2.75 | (±3.1) |  |  | 1.7 | (±2.9) | 0.9 | (±0.9) | 0.3 | (±0.8) | 0.1 | (±0.4) |
|  | **O-Y** | n=14 | 3.15 | (±2.2) |  |  | 2.3 | (±4.0) | 1.8 | (±3.0) |  |  |  |  |
| **March 2013** | **K-O** | n=9 |  |  |  |  | 0.8 | (±1.3) | 0.3 | (±1.0) |  |  | 0.3 | (±0.6) |
|  | **K-Y** | n=13 | 0.57 | (±1.0) |  |  | 0.8 | (±1.3) | 0.7 | (±1.1) |  |  |  |  |
|  | **O-O** | n=6 | 0.49 | (±0.8) |  |  |  |  | 1.0 | (±1.2) |  |  |  |  |
|  | **O-Y** | n=13 | 0.57 | (±1.0) |  |  | 1.7 | (±2.6) | 0.7 | (±1.4) |  |  | 0.1 | (±0.4) |
| **July 2013** | **K-O** | n=13 | 0.34 | (±0.6) |  |  |  |  | 0.1 | (±0.4) | 0.1 | (±0.4) |  |  |
|  | **K-Y** | n=11 | 0.8 | (±2.2) |  |  |  |  | 0.5 | (±1.0) | 0.4 | (±0.7) |  |  |
|  | **O-O** | n=13 | 0.34 | (±0.6) |  |  |  |  | 0.3 | (±0.6) | 0.3 | (±0.6) |  |  |
|  | **O-Y** | n=9 | 0.16 | (±0.5) |  |  |  |  | 0.3 | (±0.6) | 0.2 | (±0.5) |  |  |
