# Supplementary material for: Meio- and Macrofaunal Communities in Artificial Water-Filled Tree Holes: Effects of Seasonality, Physical and Chemical Parameters, and Availability of Food Resources
Source: PLoS One. 2015 Aug 18;10(8):e0133447. doi: 10.1371/journal.pone.0133447 (PMC4540321; doi:10.1371/journal.pone.0133447)
Supplement: S2 Table — K-O (Kühnauerheide, old forest), K-Y (Kühnauerheide, young forest), O-O (Oranienbaumer Heide, old forest), O-Y (Oranienbaumer Heide, young forest). (DOC) [file pone.0133447.s002.doc]

| Sample | |  |  | Biomass (µg 100cm^-2^) | | | | | | | | | | | |
| --- | --- | --- | --- | --- | --- | --- | --- | --- | --- | --- | --- | --- | --- | --- | --- |
|  |  |  |  | Nematoda | | Bdeloidea | | Tardigrada | | *Dasyhelea sp.* | | *Metriocnemus spec.* | | *Culex sp.* | |
| **May 2012** | **K-O** | n=15 |  | 3.1 | (±6.9) | 0.1 | (±0.1) | 0.004 | (±0.02) | 9.8 | (±14.1) |  |  |  |  |
|  | **K-Y** | n=15 |  | 14.4 | (±34.7) | 0.1 | (±0.2) | 0.1 | (±0.1) | 4.9 | (±16.2) |  |  |  |  |
|  | **O-O** | n=15 |  | 192.5 | (±678.0) | 0.1 | (±0.1) |  |  | 12.1 | (±21.1) |  |  |  |  |
|  | **O-Y** | n=15 |  | 73.0 | (±278.2) | 0.04 | (±0.1) |  |  | 43.8 | (±170.0) |  |  |  |  |
| **August 2012** | **K-O** | n=15 |  | 234.8 | (±441.4) | 0.8 | (±1.4) |  |  | 446.3 | (±1019.3) | |  |  |  |
|  | **K-Y** | n=15 |  | 39.5 | (±71.6) | 0.1 | (±0.0) |  |  | 542.6 | (±563.3) | 1,277.6 | (±4454.3) |  |  |
|  | **O-O** | n=15 |  | 70.1 | (±268.0) | 3.9 | (±7.9) |  |  | 336.3 | (±483.1) |  |  | 1.6 | (±6.1) |
|  | **O-Y** | n=14 |  | 317.5 | (±1096.0) | 0.1 | (±0.1) |  |  | 376.7 | (±451.1) | 8.4 | (±26.9) | 5.1 | (±19.5) |
| **November 2012** | **K-O** | n=14 |  | 528.1 | (±1544.6) | 0.3 | (±1.0) |  |  | 643.4 | (±1397.0) | |  |  |  |
|  | **K-Y** | n=15 |  | 1.0 | (±1.0) | 1.5 | (±4.0) |  |  | 257.7 | (±684.3) |  |  |  |  |
|  | **O-O** | n=15 |  | 21.5 | (±70.2) | 0.4 | (±0.7) |  |  | 487.0 | (±600.0) |  |  |  |  |
|  | **O-Y** | n=14 |  | 7.4 | (±22.3) | 3.3 | (±5.4) |  |  | 225.4 | (±406.6) |  |  |  |  |
| **March 2013** | **K-O** | n=9 |  | 18.7 | (±52.0) | 0.00 | (±0.0) |  |  | 91.7 | (±122.6) |  |  |  |  |
|  | **K-Y** | n=13 |  | 3.1 | (±9.2) | 1.7 | (±4.9) |  |  | 110.1 | (±167.5) |  |  |  |  |
|  | **O-O** | n=6 |  | 1.2 | (±2.2) | 0.01 | (±0.0) |  |  | 150.5 | (±166.6) |  |  | 0.003 | (±0.007) |
|  | **O-Y** | n=13 |  | 1.1 | (±2.0) | 0.5 | (±1.7) |  |  | 126.3 | (±282.2) |  |  |  |  |
| **July 2013** | **K-O** | n=13 |  | 110.8 | (±234.4) | 5.9 | (±15.9) | 0.04 | (±0.1) | 325.2 | (±427.9) | 0.2 | (±0.6) |  |  |
|  | **K-Y** | n=11 |  | 179.9 | (±584.0) | 154.3 | (±145.5) | |  | 435.9 | (±610.2) |  |  |  |  |
|  | **O-O** | n=13 |  | 39.4 | (±117.4) | 30.7 | (±66.9) |  |  | 471.4 | (±691.0) |  |  |  |  |
|  | **O-Y** | n=9 |  | 1.7 | (±3.1) | 35.1 | (±55.0) |  |  | 590.6 | (±517.6) | 13.3 | (±34.8) |  |  |

| Sample | |  |  | Biomass (µg 100cm^-2^) | | | | | | | | | | | | | | | | |
| --- | --- | --- | --- | --- | --- | --- | --- | --- | --- | --- | --- | --- | --- | --- | --- | --- | --- | --- | --- | --- |
|  |  |  |  | Muscidae | |  | *Psychodidae sp.* | |  | *Myathropa sp.* | |  | *Cheilosia sp.* | |  | Tabaindae | |  | Scirtidae | |
| **May 2012** | **K-O** | n=15 |  |  |  |  |  |  |  |  |  |  | 18.9 | (±37.2) |  |  |  |  | 35.4 | (±137.0) |
|  | **K-Y** | n=15 |  |  |  |  |  |  |  |  |  |  | 128.1 | (±125.1) |  |  |  |  | 88.9 | (±290.2) |
|  | **O-O** | n=15 |  |  |  |  |  |  |  |  |  |  | 42.3 | (±49.1) |  |  |  |  | 1,046.8 | (±2,723.6) |
|  | **O-Y** | n=15 |  |  |  |  |  |  |  |  |  |  | 176.2 | (±171.4) |  |  |  |  | 523.6 | (±1,314.2) |
| **August 2012** | **K-O** | n=15 |  | 971.4 | (±2,578.7) |  |  |  |  |  |  |  | 58.9 | (±101.2) |  | 15.1 | (±58.3) |  | 180.2 | (±494.2) |
|  | **K-Y** | n=15 |  | 275.4 | (±342.0) |  |  |  |  |  |  |  | 136.1 | (±344.4) |  | 4.0 | (±15.5) |  | 292.4 | (±1,132.5) |
|  | **O-O** | n=15 |  | 922.8 | (±1,733.6) |  | 247.1 | (±957.1) |  | 1,030.6 | (±3,852.8) |  | 66.4 | (±64.3) |  | 26.3 | (±68.2) |  | 336.5 | (±695.4) |
|  | **O-Y** | n=14 |  | 546.1 | (±612.1) |  |  |  |  | 2,398.9 | (±5,404.5) |  | 114.8 | (±181.3) |  | 8.0 | (±24.8) |  | 1,175.4 | (±1,866.2) |
| **November 2012** | **K-O** | n=14 |  | 48.4 | (±72.0) |  |  |  |  | 4,711.4 | (±13,243.0) |  | 16.2 | (±25.6) |  | 8.6 | (±21.8) |  |  |  |
|  | **K-Y** | n=15 |  | 58.9 | (±46.6) |  |  |  |  | 4,045.5 | (±8,943.0) |  | 61.6 | (±97.7) |  | 4.0 | (±15.5) |  | 8.0 | (±31.0) |
|  | **O-O** | n=15 |  | 56.5 | (±63.2) |  |  |  |  | 16,989.0 | (±32,701.2) |  | 34.2 | (±37.1) |  | 16.5 | (±45.2) |  | 23.3 | (±90.3) |
|  | **O-Y** | n=14 |  | 59.7 | (±44.6) |  |  |  |  | 11,540.9 | (±19,618.2) |  | 49.4 | (±78.7) |  |  |  |  |  |  |
| **March 2013** | **K-O** | n=9 |  |  |  |  |  |  |  | 17,511.0 | (±29,199.8) |  | 24.1 | (±72.3) |  |  |  |  |  |  |
|  | **K-Y** | n=13 |  | 14.9 | (±28.1) |  |  |  |  | 15,784.8 | (±26,317.0) |  | 43.6 | (±84.8) |  |  |  |  |  |  |
|  | **O-O** | n=6 |  | 14.8 | (±24.1) |  |  |  |  |  |  |  | 40.2 | (±44.8) |  |  |  |  |  |  |
|  | **O-Y** | n=13 |  | 26.0 | (±47.1) |  |  |  |  | 14,543.2 | (±22,252.1) |  | 38.6 | (±73.7) |  |  |  |  |  |  |
| **July 2013** | **K-O** | n=13 |  | 11.8 | (±25.9) |  |  |  |  |  |  |  | 6.8 | (±24.4) |  | 41.6 | (±149.9) |  | 11,515.3 | (±33,459.7) |
|  | **K-Y** | n=11 |  | 22.5 | (±58.7) |  |  |  |  |  |  |  | 17.9 | (±52.0) |  | 76.1 | (±132.3) |  |  |  |
|  | **O-O** | n=13 |  | 7.9 | (±17.1) |  |  |  |  |  |  |  | 6.0 | (±15.4) |  | 31.2 | (±60.5) |  |  |  |
|  | **O-Y** | n=9 |  | 0.2 | (±0.5) |  |  |  |  |  |  |  | 11.2 | (±21.2) |  | 7.2 | (±18.6) |  | 1,436.6 | (±5,179.6) |
