# Supplementary material for: Meio- and Macrofaunal Communities in Artificial Water-Filled Tree Holes: Effects of Seasonality, Physical and Chemical Parameters, and Availability of Food Resources
Source: PLoS One. 2015 Aug 18;10(8):e0133447. doi: 10.1371/journal.pone.0133447 (PMC4540321; doi:10.1371/journal.pone.0133447)
Supplement: S3 Table — K-O (Kühnauerheide, old forest), K-Y (Kühnauerheide, young forest), O-O (Oranienbaumer Heide, old forest), O-Y (Oranienbaumer Heide, young forest); * Because of the low water level the probes could only be used in single cups such that a mean could not be determined. Chl-a, chlorophyll-a; AFDM, ash-free dry mass. (DOC) [file pone.0133447.s003.doc]

| Sample | |  | O2 (%) | | Conductivity (µS/cm) | | pH | | Water (ml) | | Chla (µg/cm²) | | AFDM (mg/cm²) | | Last rain (days) | Last frost (days) | Average daily rain (l/m²) | | Average daily temperature (°C) | |
| --- | --- | --- | --- | --- | --- | --- | --- | --- | --- | --- | --- | --- | --- | --- | --- | --- | --- | --- | --- | --- |
| **May 2012** | K-O |  |  |  |  |  |  |  | residual moisture | | 3.2 | (±3.3) | 3.8 | (±1.7) | 15 | 53 | 0.6 | (±1.8) | 12.1 | (±5.5) |
|  | K-Y |  | 35.0 | * | 437.3 | (±30.7) | 6.9 | (±0.1) | 45.3 | (±94.4) | 3.0 | (±1.4) | 7.5 | (±4.1) |  |  |  |  |  |  |
|  | O-O |  | 37.8 | * | 679.0 | * | 6.9 | * | 33.7 | (±73.6) | 3.9 | (±1.3) | 6.4 | (±3.4) |  |  |  |  |  |  |
|  | O-Y |  | 28.3 | * | 835.0 | * | 6.6 | * | 17.7 | (±68.4) | 2.3 | (±1.1) | 7.5 | (±3.3) |  |  |  |  |  |  |
| **August 2012** | K-O |  | 30.8 | (±24.7) | 361.6 | (±228.3) | 7.0 | (±0.3) | 490.1 | (±105.3) | 29.1 | (±12.3) | 11.8 | (±5.8) | 2 | 121 | 2.1 | (±3.8) | 17.8 | (±3.8) |
|  | K-Y |  | 76.5 | (±18.8) | 186.8 | (±109.3) | 6.1 | (±0.6) | 385.5 | (±117.7) | 18.7 | (±13.9) | 16.7 | (±5.7) |  |  |  |  |  |  |
|  | O-O |  | 79.2 | (±13.4) | 144.6 | (±60.1) | 6.8 | (±0.7) | 523.2 | (±116.9) | 29.4 | (±10.2) | 20.4 | (±5.3) |  |  |  |  |  |  |
|  | O-Y |  | 35.8 | (±19.5) | 342.1 | (±94.9) | 6.5 | (±0.8) | 257.6 | (±114.4) | 13.8 | (±9.4) | 18.4 | (±5.2) |  |  |  |  |  |  |
| **November 2012** | K-O |  | 28.6 | (±14.0) | 290.3 | (±328.6) | 5.9 | (±0.5) | 100.0 | (±124.1) | 27.4 | (±9.7) | 8.0 | (±5.1) | 1 | 7 | 0.8 | (±1.7) | 13.4 | (±5.2) |
|  | K-Y |  | 20.5 | (±15.5) | 284.3 | (±69.3) | 6.6 | (±0.4) | 200.0 | (±248.9) | 20.2 | (±24.7) | 15.0 | (±8.4) |  |  |  |  |  |  |
|  | O-O |  | 22.3 | (±14.9) | 279.0 | (±69.2) | 6.7 | (±0.4) | 166.0 | (±190.8) | 23.3 | (±8.1) | 10.7 | (±5.3) |  |  |  |  |  |  |
|  | O-Y |  | 12.7 | (±11.6) | 353.8 | (±109.7) | 6.8 | (±0.1) | 34.3 | (±68.9) | 12.8 | (±9.5) | 13.4 | (±4.7) |  |  |  |  |  |  |
| **March 2013** | K-O |  |  |  | 231.1 | (±69.4) | 7.1 | (±0.9) | 659.4 | (±93.8) | 4.9 | (±3.2) | 18.7 | (±14.9) | 6 | 0 | 1.1 | (±2.0) | 1.3 | (±4.3) |
|  | K-Y |  |  |  | 414.5 | (±627.8) | 7.6 | (±0.9) | 578.1 | (±109.2) | 3.7 | (±1.4) | 15.9 | (±6.0) |  |  |  |  |  |  |
|  | O-O |  |  |  | 155.6 | (±53.8) | 7.1 | (±0.9) | 641.7 | (±145) | 5.1 | (±2.0) | 7.0 | (±2.3) |  |  |  |  |  |  |
|  | O-Y |  |  |  | 187.7 | (±25.1) | 6.9 | (±0.5) | 447.7 | (±326.5) | 3.0 | (±0.8) | 10.7 | (±5.2) |  |  |  |  |  |  |
| **July 2013** | K-O |  | 49.2 | (±12.8) | 132.6 | (±103.7) | 7.2 | (±1.1) | 543.1 | (±66.5) | 64.6 | (±42.7) | 16.9 | (±11.8) | 1 | 92 | 2.1 | (±5.4) | 13.1 | (±5.8) |
|  | K-Y |  | 37.9 | (±13.8) | 106.8 | (±41.3) | 7.1 | (±0.4) | 572.7 | (±112.5) | 40.9 | (±19.7) | 43.5 | (±27) |  |  |  |  |  |  |
|  | O-O |  | 53.3 | (±12.5) | 97.4 | (±26.7) | 7.6 | (±1.0) | 599.2 | (±166.9) | 48.8 | (±18.3) | 23.0 | (±6.2) |  |  |  |  |  |  |
|  | O-Y |  | 45.4 | (±23.2) | 274.7 | (±78.5) | 7.4 | (±0.1) | 427.8 | (±234) | 54.2 | (±29.9) | 18.3 | (±7.0) |  |  |  |  |  |  |
